# Surface modification of polypropylene nonwoven with chitosan, TiO_2_, and Ag nanoparticles for the removal of acid dye from water

**DOI:** 10.55730/1300-0527.3517

**Published:** 2022-12-12

**Authors:** Aminoddin HAJI, Mohsen HADIZADEH, Masoumeh FERDOSI

**Affiliations:** Department of Textile Engineering, Yazd University, Yazd, Iran

**Keywords:** Photocatalyst, nanoparticle, functionalization, plasma treatment

## Abstract

In this study, polypropylene nonwoven was coated with chitosan after being activated by oxygen/argon low-pressure plasma. The chitosan-treated sample (PP-Ch) was coated with TiO_2_ nanoparticles by a pad-dry method. Silver nanoparticles were in situ synthesized on the chitosan-TiO_2_-coated sample by a chemical reduction method. The morphology of each of the samples mentioned above was evaluated by FESEM. The efficiency of each sample in the removal of C.I. Acid Brown 248 from aqueous media was evaluated. The PP-Ch sample removed 90% of the dye at pH=3. Coating the PP-Ch sample with TiO_2_ and silver nanoparticles improved the dye removal efficiency under UV irradiation.

## 1. Introduction

Polypropylene (PP) nonwoven is vastly employed in applications such as air filtration, face masks, liquid filtration, thermal insulation, oil-absorbing materials, wiping cloth, etc. PP nonwoven has several advantages such as durability, low thermal conductivity, low density, resistance to chemicals, sunlight, and microorganisms, besides low cost. However, this polymer contains no chemically active group, which restricts its applications in some cases. Various modification techniques, e.g., plasma treatment, grafting, and UV irradiation, have been employed for the functionalization and improvement of different properties of PP nonwovens [1–3]. Plasma treatment creates free radicals on the surface of the PP fibres, which act as active sites for grafting different monomers and polymers on it [4].

Marković et al. employed corona discharge to activate PP nonwoven to attach sodium alginate on its surface. Copper ions were then adsorbed on the carboxylate ions of the sodium alginate and reduced to form copper oxide nanoparticles. The finished nonwoven showed excellent antimicrobial activity [5]. Erben et al. employed DBD plasma for activation of PP nonwoven and applied a chitosan coating on the plasma-activated PP fibres. The chitosan-coated PP nonwoven exhibited antibacterial and antibiofouling properties [6].

Functionalization of PP nonwoven is one of the widely studied approaches for the preparation of adsorbents for the removal of various kinds of pollutants from water and air. Wei et al. used low-pressure plasma treatment for activation of PP nonwoven and grafting of chitosan and 1-vinylimidazole on it. The prepared adsorbent removed Cu^2+^ from water efficiently [7]. Haji et al. employed oxygen plasma as a pretreatment to initiate the grafting of acrylic acid on PP nonwoven and employed the acrylic acid-grafted nonwoven for the removal of cationic dye from water [8, 9]. Attachment of poly(propylene imine) dendrimer on the acrylic acid-grafted nonwoven granted the ability to remove anionic dyes from water as well [3].

In another study, chitosan was grafted on the plasma-treated acrylic acid-grafted PP nonwoven, and the resultant adsorbent showed the ability to trap copper ions from water [10].

Other studies revealed that polypropylene films coated with TiO_2_ nanoparticles showed photocatalytic activity under visible light and degraded Alizarin Red S in aqueous media [11]. Several investigations confirmed that Ag nanoparticles embedding on TiO_2_-based photocatalyst improved its photocatalytic activity for degradation of organic pollutants such as dyes and extended its photocatalytic activity to visible light [12–14].

In this study, polypropylene nonwoven was activated by oxygen/argon plasma, and chitosan was grafted on the activated surfaces. Then, TiO_2_ and Ag nanoparticles were deposited on its surface, respectively. Finally, the removal of an acid dye by the nonwoven at each coating stage was investigated.

## 2. Materials and methods

PP spunpond nonwoven with a density of 20 g/m^2^ was obtained from Baftineh CO., Iran, and scoured with a nonionic surfactant before use. Medium molecular weight chitosan and silver nitrate were obtained from Merck, Germany. Titanium Dioxide nanoparticles with average particle size of 15–25 nm were obtained from Tecnan, Spain.

Chitosan coating: plasma treatment was done on a piece of low-pressure equipment made by Basafan, Iran. A mixture of oxygen and argon was used as the processing gas, while the flow rate of each gas was 50 Sccm. Plasma treatment was done for 10 min with a power of 150 W. The plasma-treated samples were immediately immersed for 5 min in a solution containing 1% chitosan and 1% acetic acid. Finally, the samples were padded with the pressure of 2 bar and dried in a stenter at 80 °C for 5 min.

TiO_2_ coating: chitosan-coated samples (PP-Ch) were immersed in dispersions of TiO_2_ with different concentrations (0.5%–2%) under sonication (550 W) for 15 min, then dried in a stenter at 80 °C for 10 min (PP-Ch-TiO_2_).

Silver nanoparticle coating: PP-Ch-TiO_2_ samples were immersed in silver nitrate solution (200 ppm) for 10 min, and then transferred to a solution of sodium borohydride (400 ppm) for 10 min to reduce the silver ions to silver nanoparticles (PP-Ch-TiO_2_-Ag).

Dye removal studies: 5 solutions of C.I. Acid Brown 248 in distilled water (100 mg/L) were prepared at different pH values ranging from 1 to 10. To assess the dye removal efficiency of PP-Ch at each pH value, 0.1 g of the adsorbent was immersed in 25 mL of the dye solution, and the absorption of the solution was measured in different time intervals (up to 120 min) using a UV-Vis spectrophotometer (EU-220, Onlab, China) at 387 nm. The dye removal efficiency of the nanoparticle-coated samples was measured by the same procedure under UV irradiation (UVC, 400 W).

Surface morphology: field emission scanning electron microscope (Mira3, Tescan, Czech Republic) was employed to study the surface morphology of the samples after each modification stage.

## 3. Results

**Figure 1:**
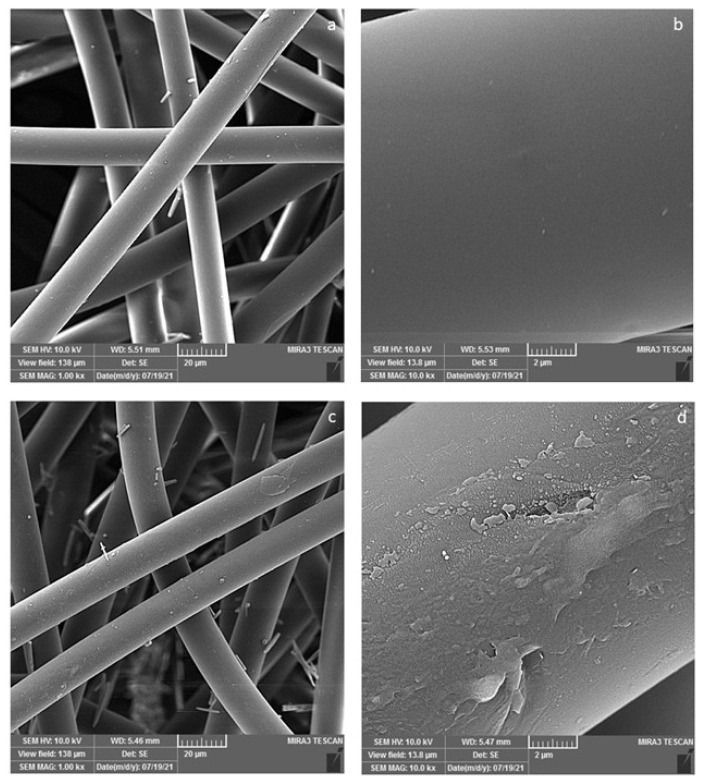
FESEM images of raw (a, b) and plasma-treated (c, d) PP fibres.

Figure 1 shows the FESEM images of the raw and plasma-treated samples at two magnifications. The surface of the raw fibres is smooth. It is evident that plasma treatment created some etching and increased the surface roughness due to the bombardment of the fibres with high-energy species. Furthermore, oxygen-containing groups are created on the surface of PP fibres after plasma treatment which has been approved in previous studies [2]. These groups are responsible for the attachment of chitosan on the surface of PP fibres.

**Figure 2:**
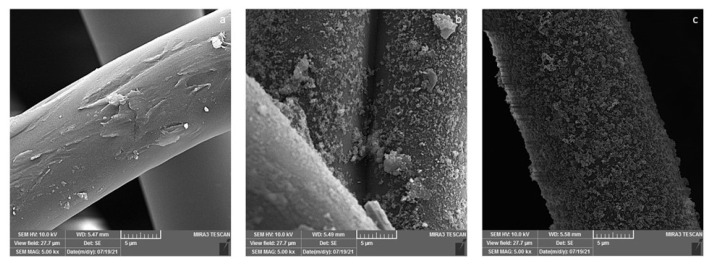
FESEM images of PP-Ch (a), PP-Ch-TiO_2_ (b), and PP-Ch-TiO_2_-Ag (c).

Coating of PP fibres with chitosan biopolymer can be seen in Figure 2a, and the presence and uniform distribution of the titanium dioxide and silver nanoparticles can be seen in Figures 2b and 2c as well. EDX images were also taken (not shown here), which confirmed that the titanium dioxide and silver nanoparticles are present on the surface and well distributed.

**Figure 3:**
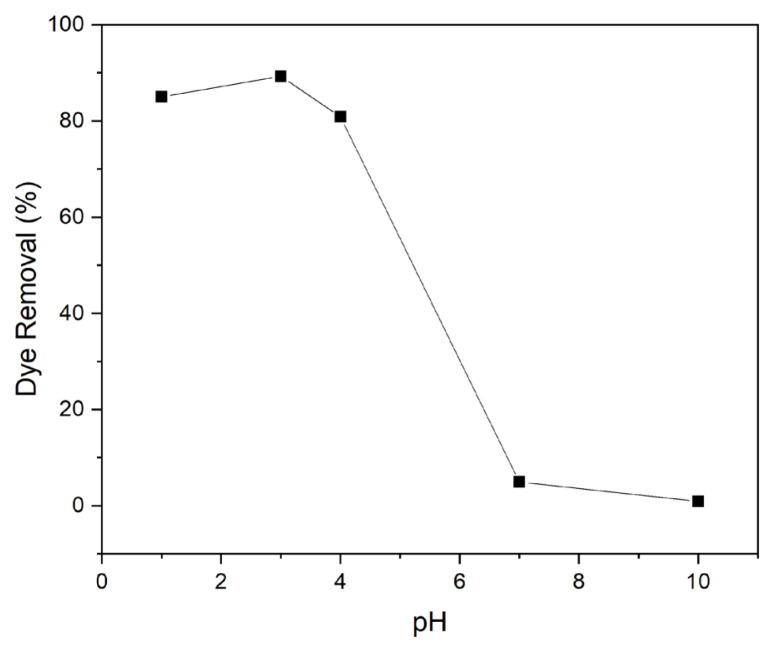
The effect of pH on dye removal by PP-Ch.

Figure 3 shows the percent dye removal by PP-Ch at various pH levels. It can be seen that the maximum dye removal took place at pH = 3 which is due to the protonation of the amine groups of chitosan and the electrostatic attraction between the dye anions and the positive charges on the modified fibres [15].

**Figure 4:**
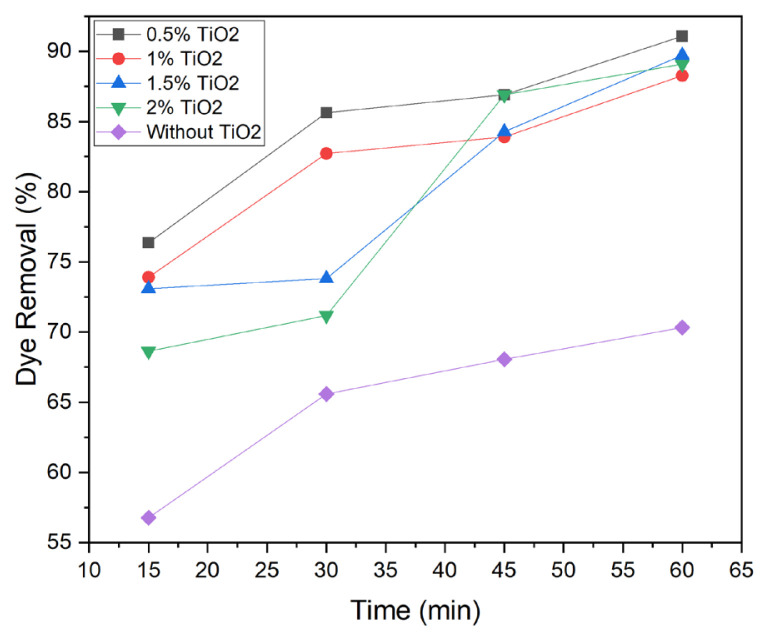
Effect of time on dye removal efficiency of samples with various TiO_2_ loading under UV irradiation.

The change of percent dye removal by PP-Ch-TiO_2_ samples containing different amounts of TiO_2_ nanoparticles under UV radiation during times ranging from 15 to 60 min is shown in Figure 4. The dye removal efficiency increased by increasing the exposure time.

**Figure 5:**
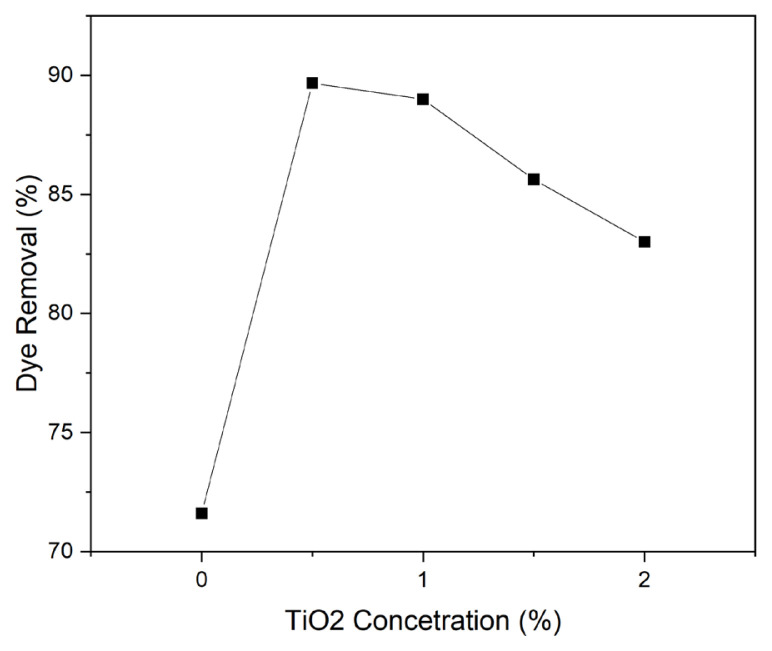
Effect of TiO_2_ loading on dye removal efficiency of PP-Ch-TiO_2_ samples after 60 min.

Figure 5 shows the dye removal efficiency of the samples after 60 min of UV irradiation. It can be seen that the optimum amount of TiO_2_ nanoparticles loading was 0.5%, and the dye removal efficiency was decreased at higher TiO_2_ nanoparticles loading, which may be due to the agglomeration of the nanoparticles on the surface and avoiding the accessibility of the dye molecules to the adsorbent sites. The fundamental mechanisms of TiO_2_ photocatalytic activity have been discussed in many studies [13].

**Figure 6:**
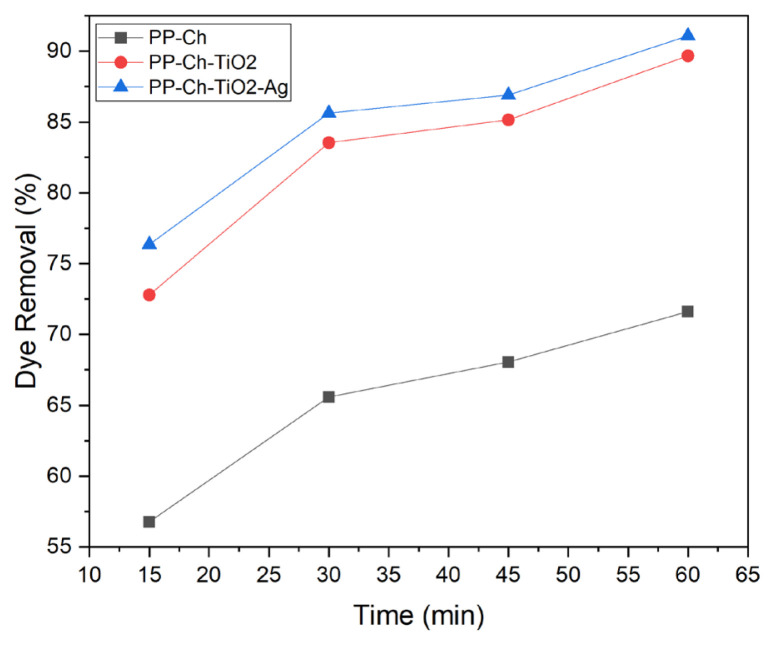
Comparing the dye removal efficiency of PP-Ch, PP-Ch-TiO_2_, and PP-Ch-TiO_2_-Ag samples under UV irradiation after 60 min.

Figure 6 compares the chitosan-coated sample with the samples coated with optimum amounts of TiO_2_ and silver nanoparticles. It can be seen that the coating with TiO_2_ nanoparticles increased the dye removal efficiency under UV irradiation considerably, due to the photocatalytic activity of these nanoparticles. Adding silver nanoparticles on the surface of the PP-Ch-TiO_2_ sample made a slight improvement in the dye removal efficiency. According to the literature, silver nanoparticles possess some photocatalytic activity. Furthermore, the presence of silver nanoparticles enhances the TiO_2_ photocatalytic activity by trapping the electrons transferred from the conduction band of the TiO_2_ semiconductor and transferring these electrons to oxygen which converts them to superoxide radicals which decompose the dye molecules. The surface plasmon resonance of silver nanoparticles extends the light absorption to the visible light region and enhances the photocatalytic activity of TiO_2_ nanoparticles under visible light [13, 14].

## 4. Conclusion

PP nonwoven was functionalized by plasma, chitosan, TiO_2_, and silver nanoparticles, respectively. The ability of the modified samples for the removal of an anionic dye from the water was studied. The PP-Ch sample removed about 90% of the dye at pH = 3. The application of TiO_2_ and Ag nanoparticles on the surface of PP-Ch enabled the adsorbent to degrade the dye molecules under UV irradiation, and the dye removal efficiency was improved. The prepared photo-active adsorbent can be a good candidate for adsorptive and photocatalytic removal of anionic dyes from textile wastewater.
